# What is the power of a genomic multidisciplinary team approach? A systematic review of implementation and sustainability

**DOI:** 10.1038/s41431-024-01555-5

**Published:** 2024-02-20

**Authors:** Alan Ma, Rosie O’Shea, Laura Wedd, Claire Wong, Robyn V Jamieson, Nicole Rankin

**Affiliations:** 1 Specialty of Genomic Medicine, University of Sydney, Sydney, NSW Australia; 2 Department of Clinical Genetics, Children’s Hospital at Westmead, The Sydney Children’s Hospitals Network, Sydney, NSW Australia; 3 Eye Genetics Research Unit, Children’s Medical Research Institute, Sydney, NSW Australia; 4 Melbourne School of Population and Global Health, University of Melbourne, Melbourne, VIC Australia; 5 Sydney School of Public Health, University of Sydney, Sydney, NSW Australia

**Keywords:** Medical genomics, Genetic testing

## Abstract

Due to the increasing complexity of genomic data interpretation, and need for close collaboration with clinical, laboratory, and research expertise, genomics often requires a multidisciplinary team (MDT) approach. This systematic review aims to establish the evidence for effectiveness of the genomic multidisciplinary team, and the implementation components of this model that can inform precision care. MEDLINE, Embase and PsycINFO databases were searched in 2022 and 2023. We included qualitative and quantitative studies of the genomic MDT, including observational and cohort studies, for diagnosis and management, and implementation outcomes of effectiveness, adoption, efficiency, safety, and acceptability. A narrative synthesis was mapped against the Genomic Medicine Integrative Research framework. 1530 studies were screened, and 17 papers met selection criteria. All studies pointed towards the effectiveness of the genomic MDT approach, with 10-78% diagnostic yield depending on clinical context, and an increased yield of 6-25% attributed to the MDT. The genomic MDT was found to be highly efficient in interpretation of variants of uncertain significance, timeliness for a rapid result, made a significant impact on management, and was acceptable for adoption by a wide variety of subspecialists. Only one study utilized an implementation science based approach. The genomic MDT approach appears to be highly effective and efficient, facilitating higher diagnostic rates and improved patient management. However, key gaps remain in health systems readiness for this collaborative model, and there is a lack of implementation science based research especially addressing the cost, sustainability, scale up, and equity of access.

## Introduction

The genomic era has expanded the availability of diagnostic testing, management and options for precision care for many genetic conditions. Diagnostic yields have improved by 40-80% depending on the condition, unlocking new genetic diagnoses, family planning options and access to advanced therapeutics including gene therapies [1]. The publication of the American College of Medical Genetics (ACMG) guidelines on variant interpretation [2], brought about increased uniformity in laboratory reporting of results, and efforts to enhance pathogenicity calling and reduce the burden of variants of uncertain significance (VUS).

Much of the current challenge is in the interpretation of novel variants and genes, assigning pathogenicity and clinical correlation to a multitude of scenarios, often requiring inter-disciplinary expertise in molecular, clinical, functional genomics, as well as in organ-specific areas such as oncology, cardiology, nephrology, ophthalmology, and neurology. In addition, several cutting-edge applications of genomics such as advanced therapeutics, prenatal and acute care genomics, require additional expertise and close collaboration and liaison between clinical and, laboratory staff, as well as researchers, in order to maximise the benefits of genomic testing and diagnosis. Due to this complexity and knowledge-specific requirements, multi-disciplinary genomic teams (hereafter ‘MDT’), have been increasingly utilized and recommended in a number of guidelines [3, 4]. This follows the example of other similar complex team approaches in disciplines such as oncology.

However, despite many recommendations for a genomic MDT approach in guidelines and position statements, there remains a paucity of evidence about ideal approaches to multidisciplinary care in the genomics field. Due to the rapidity of advances in genomic/precision medicine, evidence-based models on the ‘best practice’ approach for the distinct and complex needs of genomic medicine do not yet exist. International studies describe an insufficient genomics workforce to meet demand [5, 6], and models of engaging highly specialized clinicians and scientists in yet more meetings and discussions raises whether the MDTs are the most effective and efficient use of limited time and resources. In addition, few studies have evaluated the characteristics and factors that promote effective genomic MDTs and their impact on patient, health service, and implementation level outcomes, such as acceptability, feasibility, adoption and sustainability [7].

A wealth of literature exists in the non-genetics fields regarding the success and effectiveness of MDT models [8]. MDTs have demonstrated improved outcomes in increasingly complex health care systems and are widely accepted as ‘gold standard’ in cancer care delivery worldwide. By harnessing the combined expertise of disciplines including surgeons, oncologists, radiologists, pathologists, nurses and physicians, to meet and discuss complex cancer care, optimal management plans and pathways are utilized and this is now standard of care in cancer [9]. Further research into effective cancer MDT practices have highlighted the importance of team relationship, communication, leadership, inclusiveness, and careful consideration of patient and psychosocial issues in team decision making processes [8], although key evidence-practice gaps still exist, highlighting the need for more implementation research in this field [10].

Implementation research is especially needed in the rapidly developing field of genomics [1] and its application to clinical practice, such as the uptake of MDT approaches. Without an evaluation of the key implementation factors, the evidence for genomic MDTs as an effective intervention and potential for adaptability and scalability is lacking. This review aims to use an implementation science framework to examine the core components of the genomic MDT that achieve a diagnostic rate and inform clinical care for patients undergoing genomic testing. Using implementation frameworks can improve the study of interventions such as MDTs, to understand the health services factors and outcomes that promote uptake and successful implementation [7]. The Genomic Medicine Integrative Research [GMIR] framework [11] was designed by the genomics community and ‘Implementing Genomics into Practice’ [IGNITE] consortium [12] by adopting the well-used implementation Consolidated Framework for Implementation Research [CFIR] constructs [13, 14] into the genomic context. It has been utilized in implementation research as an adaptable tool for evaluating the clinical implementation of genomic programs [15]. GMIR garners broad evidence of context, process, interventions and outcomes of such programs to understand sustainability.

O*ur primary review question is: How effective is the coordinated genomic multidisciplinary care approach, in facilitating genetic diagnostics and precision medicine*?

*Our secondary review questions are: What are the key implementation components and outcomes of the genomic multidisciplinary care model? What are the evidence gaps and determinants of practice that can inform a model of multidisciplinary care in genomics?*

## Methods

**Fig. 1:**
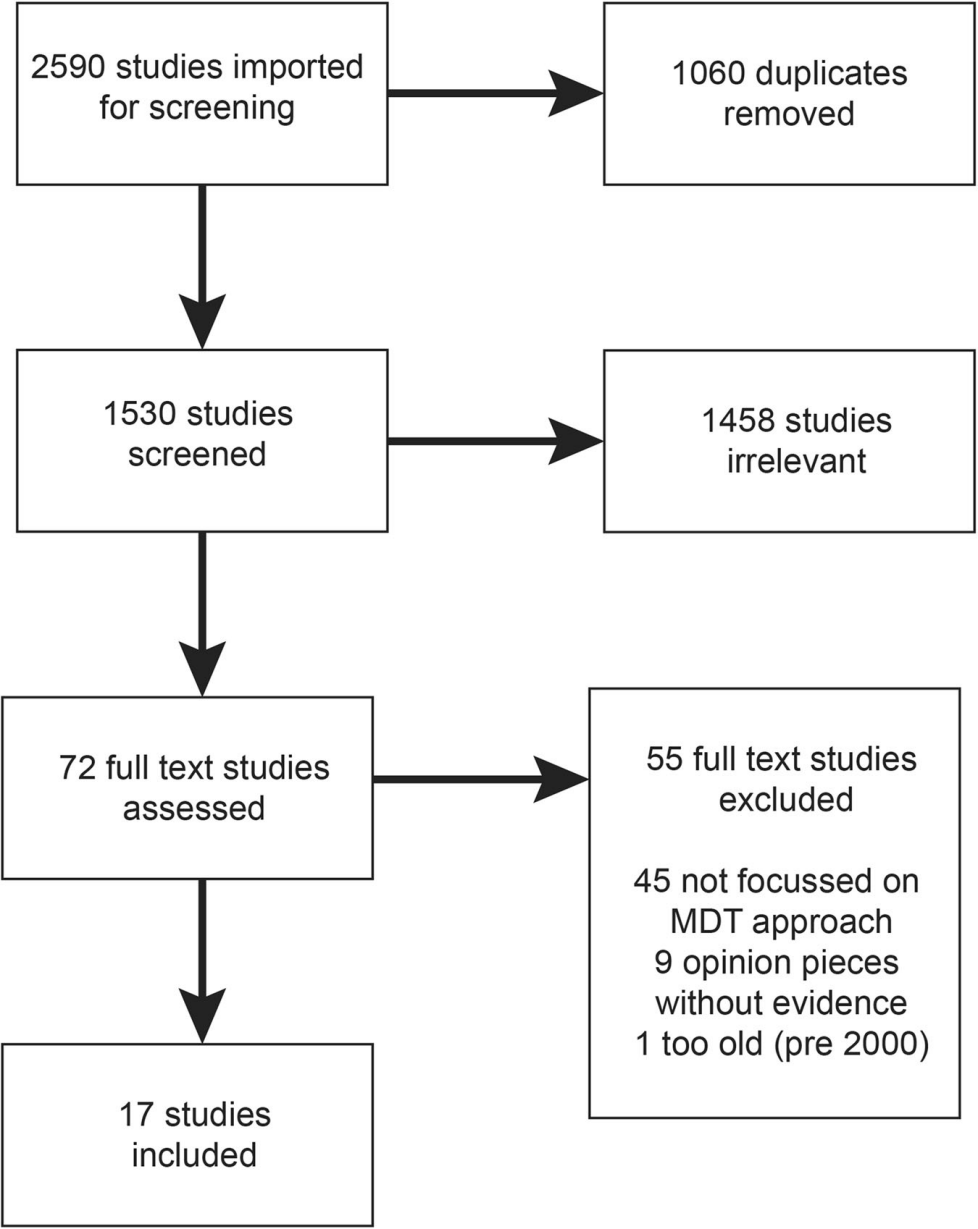
PRISMA flow diagram. Flow diagram demonstrating the screening, removal, and selection of studies in this review, with reasons.

The findings of this study have been reported in line with Preferred Reporting Items for Systematic reviews and Meta-Analyses (PRISMA) statement (Fig. 1**)** [16]. The study protocol was registered on PROSPERO (https://www.crd.york.ac.uk/prospero/ ID CRD42022373661) on 17^th^ November 2022.

### Searches

We searched MEDLINE, Embase, PsycINFO databases for papers published after 2010 that were in the English language. Search terms (Supp. Table 1) were developed according to the Population, Intervention, Comparison and Outcome (PICO) with MESH and Emtree terms, focussing on the genomic literature with an interdisciplinary/multidisciplinary/shared decision making approach to care and communication. A preliminary search in November 2022 was followed by a repeat search once data extraction was complete in March 2023, to identify any new publications.

This systematic review seeks to evaluate the effectiveness of a coordinated genomic multidisciplinary team approach, which incorporates genetic/genomic expertise, medical subspecialists, and laboratory/scientific involvement for providing genomic diagnostics and medical management of genetic conditions.

The ***population*** consisted of both: patients undergoing genomic testing in a medical setting for diagnosis, and the clinicians referring these patients for genomic diagnostic opinion and management.

The ***intervention*** is a coordinated multidisciplinary care approach to genomics, which involved a close collaboration (either virtual or in person via meetings) of both:

Genetics/genomics expertise (usually clinical geneticists, molecular genetics laboratory scientists and genetic counsellors), identifying genetic diagnoses (genotyping) for patients, and

Subspecialist clinicians and subject matter experts (from varied disciplines) working in conjunction with genomics to identify clinical diagnoses (phenotyping) and management implications

The search ***comparator/control:*** In comparison to the coordinated multidisciplinary approach to genomic diagnostics, the main alternatives include ‘standard care’ via individual clinicians, laboratories and genetics services. Many studies (especially qualitative) may not include a comparator or control group.

Main ***outcomes*** reported by the study include:

Evidence on the effectiveness of the genomic multidisciplinary care approach, in facilitating genetic diagnostics and precision medicine and management outcomes

Clinical Service level outcomes such as efficiency, safety, equity, timeliness

Implementation outcomes including acceptability, adoption, appropriateness, cost, feasibility, fidelity, penetration, sustainability

### Types of study included

We included qualitative and quantitative studies of the genomic MDT approach, including observational and cohort studies. Purely descriptive studies were included if they were primarily describing a coordinated multidisciplinary team approach to genomic diagnostics and precision care, the focus of this review.

Studies were excluded where they did not demonstrate a focus on the coordinated MDT approach to genomic diagnostics and precision medicine, such as:

1. Studies that focussed mainly on the genetic counselling, clinical management or surgical decision-making aspects without addressing the genomic diagnostic issues
2. Papers that were conference proceedings, case reports, systematic reviews, editorial or commentaries.

#### Study selection

Covidence (www.covidence.org) was utilized to import all studies for abstract screening by three authors against the key inclusion and exclusion criteria.

The criteria were initially piloted by two authors for the first 20 articles, then further refined after discussion of discrepancies, and adjudication with a third author.

Studies that initially met the selection criteria were retained for eligibility checks by two authors and all reasons for excluding were documented in Covidence. Once authors reached consensus on screened abstracts, these proceeded to full text review. Two authors conducted the full text review to ensure consistency according to the above criteria, and final papers were selected for data extraction. Any conflicts were resolved by discussion and cross-checking with a third author.

### Data extraction

A data extraction form was developed by one author, and refined in consultation with the study team. Data was extracted for the following fields: (Supp. Table 2) population, intervention (adapted criteria from TIDieR checklist [17] and STARI guidelines[18]), and use of implementation framework, study design, setting, and intervention outcomes mapped to Proctor et al.’s evaluative framework outcomes at the service and implementation levels [7].

Proctor’s evaluative framework consists of healthcare service (Efficiency, safety, effectiveness, equity, patient centeredness, timeliness) and implementation (acceptability, adoption, appropriateness, cost, feasibility, fidelity, penetration, sustainability) outcomes. These are well-used constructs in the implementation science literature since 2011, in over 400 papers as a standard measure of implementation outcomes [19]. These measures, and their definitions in the genomic MDT context, were further characterized by GMIR domain mapping to understand the genomic MDT outcomes (Supp table S2) by two authors prior to extraction.

While Proctor et al. provide a comprehensive overview of service and implementation level factors, the GMIR framework was used for its specific application in the genomics field. In particular, it takes into account the contextual (system and clinician), interventional, process, and broader outcomes (health policy and economic utility) factors relevant to genomic medicine.

Two authors independently extracted the data from 20% of studies each, selecting different study designs for consistency across types, and compared notes to minimize bias and improve accuracy, with discrepancies resolved via discussion with a third author. Once consistent extraction was achieved, one author completed data extraction for the remaining 60% of articles.

### Risk of bias/quality

As both qualitative and quantitative studies were included, the QualSyst Assessment Tool [20] was used to assess quality. One author assessed all studies for risk of bias, and a second author assessed 50% of the studies, and discrepancies in scoring were resolved by discussion and third author for resolution.

### Strategy for data synthesis

A narrative synthesis was performed using both Proctor [7] and GMIR [11]. The first step in the synthesis was based on the Proctor et al. evaluative framework outcomes [7] at service, and implementation level and further subthemes developed from this structure (Tables S2 and S3). This was performed by one author, and further correlated by a second. Where possible, quantitative data on the effectiveness of the MDT approach were collected. Further analysis of implementation outcomes and evidence gaps were mapped to the GMIR framework [11], which is based on the Consolidated Framework for Implementation Research (CFIR) [13]. This was used to facilitate an understanding of the processes and core components of genomic MDTs as defined at the contextual, intervention, process and outcomes levels (Supp. Table S3). The GMIR provides a simple, clear, comprehensive framework for genomic research, interventions, and understanding processes that influence implementation. The narrative synthesis combined the summary and explanation of the intervention characteristics and potential effects.

## Results

A total of 2590 studies were imported to Covidence for screening (Fig. 1). Duplicates (*n* = 1060) were removed, leaving 1530 studies to be screened, of which 72 were agreed upon for full text review. A total of 55 studies were excluded, and 17 papers met the selection criteria. Of the 17 genomic MDT papers, five were qualitative, two were mixed methodology, and the remaining ten were mainly descriptive cohort studies with quantitative elements (Tables 1, 2, and Supplementary Table S3).

**Table 1:** Study characteristics of genomic MDT papers in this review.

Surname and year	Location and Context	Type of genomic MDT studied	Type of study	Qualsyst Score
Ormonroyd 2017 [29]	UK—NHS referrals for rare disease genomics	Rare disease	Qualitative—interviews of 19 genomic MDT members	0.5
Fishler 2019 [30]	USA—single site	Cancer	Qualitative—interviews of 12 members of cancer MDT	0.45
Mancini 2021 [22]	Netherlands—referral centre	Brain malformation	Descriptive case vignettes with some qualitative elements	0.2
Lynch 2020 [31]	Australia—12 tertiary hospital sites	Acute neonatal	Qualitative—interviews of 16 genetic counsellors	0.75
Hill 2020 [32]	UK—single site tertiary referral	Acute paediatric	Qualitative—interviews of 19 HCP and 11 parents	0.7
Vadlamudi 2021 [25]	Australia—3 tertiary hospital sites	Epilepsy	Mixed methods Qualitative and quantitative review of cohort including MDT	0.45 qual 0.5 quant
Taylor 2019 [41]	UK—national genomic service	Rare disease	Prospective cohort study and quantitative review of 132 patients discussed in MDT	0.5
Lazaridis 2016 [23]	USA—Mayo clinic	Rare disease	Descriptive retrospective study with quantitative review of 82 patients referred for testing	0.46
Marinakis 2021 [24]	Greece—national referral service	Rare disease	Descriptive retrospective cohort study with quantitative review of 400 patients referred for testing	0.5
Jayasinghe 2021 [27]	Australia—4 tertiary sites	Renal	Prospective multicentre study with quantitative review of 224 patients referred to 4 MDT sites	0.5
Mallett 2017 [28]	Australia—single site	Renal	Descriptive retrospective study with quantitative review of 140 patients referred to renal MDT laboratory	0.5
Lunke 2018 [21]	Australia—2 tertiary sites	Acute paediatric	Mixed method: prospective evaluation with qualitative and quantitative review of 40 patients referred at 2 sites, with implementation framework (CFIR)	0.45 qual 0.6 quant
Mone 2019 [35]	UK—tertiary referral site	Foetal/ Prenatal	Retrospective cohort study with quantitative review of 256 patients referred to prenatal MDT	0.5
Chandler 2018 [36]	UK—Skeletal prenatal referral site	Foetal/ Prenatal	Retrospective cohort study with quantitative review of 19 cases referred to prenatal skeletal dysplasia MDT	0.46
Petrovsky 2019 [46]	USA—tertiary referral service	Foetal/ Prenatal	Prospective cohort study with quantitative review of 517 cases referred to prenatal MDT	0.5
Cornthwaite 2022 [34]	Canada—single site referral centre	Foetal/ Prenatal	Quantitative review of 90 patients referred for foetal MDT	0.5
Rupp 2019 [26]	Germany—single site referral centre	Cardiac	Prospective cohort study with Quantitative review of 42 patients referred to cardiac MDT	0.5

**Table 2:** Mapping the genomic MDT characteristics to the GMIR framework and Proctor outcomes.

GMIR	GMIR subdomain	Narrative synthesis and proctor outcomes	Quotes
Contextual factors	Healthcare systems utilising MDTs	Genomic MDTs were in tertiary centres and expertise settings, for highly specialised complex cases in prenatal, renal, neurology, cardiology, cancer, undiagnosed disease, acute paediatric settings.	
	Social Determinants of MDT	Western countries, mostly English speaking, with funding/insurance available for genomic testing in Europe, UK, USA, Australia	
	Clinician Factors in MDT	Clinicians with genomic literacy an important factor in engagement with genomics and laboratory staff and use of MDT resources	
Intervention	Genomic Yield of MDT	**Effectiveness**—the genomic MDT approach was effective with a diagnostic yield of: • Undiagnosed disease: 31.6% [41], 29% [23], 53% [24] with increased diagnostic rate of 3.7% [22] to 6.6% [41] from interdisciplinary interaction, and detailed phenotype driven variant filtration [24] • Epilepsy: 17%, with 92% having impact on management [25] • Renal: 39% [27] and 43% [28] with better testing triage, interpretation of VUS [28], and management implications in 59% [27] • Acute Paediatrics: 42% [32], 52.5% [21], altering management in 57% [21] • Prenatal: 47.7% [35], 81% [36], 10% [46], 23% [34]with 6% increased due to MDT review of VUS [34] • Cardiac: 78%, with an additional 9 (25%) cases solved based on MDT discussion and re-examination [26]	*‘Diagnostic yield and clinical utility….are highly dependent on the close collaboration of a multidisciplinary team’* [24] *Because of the broad differential diagnosis in childhood, a multidisciplinary assessment is recommended for further outcome studies for patients with HCM*. [26] *‘All professional groups valued cross-discipline collaborative working when delivering RGS, highlighting the benefits of multidisciplinary team meetings to triage patients and interpret results.’* [29]
	Genomic MDT Characteristics	**Efficiency** – The MDT was an efficient use of resources due to: • Collaboration which led to diagnosing and managing complex patients [41] and collaborative processes for a rapid turnaround [31], more appropriate case selection, discussion amongst specialists for shared decision making and genomic education [29, 36] • VUS resolution: reclassification of VUS with MDT discussion of clinical context [23], reduction in VUS and false positives requiring additional time/analysis [24], reduction in number of variants requiring curation by 2/3 [25], better curation of panels and variant interpretation [28] **Appropriateness**: the genomic education level of the referring subspecialists also had an impact on the MDT utility, in terms of appropriate case selection for testing and variant interpretation [27]	*‘the complexity of interpreting genomic results in the prenatal context with incomplete phenotyping requires close collaboration and information sharing between the clinical team and the clinical laboratories.’* [34]
Processes	Healthcare system processes in MDT	**Safety** of the MDT was highlighted, especially in: • VUS resolution: which is problematic in prenatal situation. VUS rate ranged from 7-33% depending on lab, including some labs with multiple VUS reported. MDT was vital for resolving these issues and guiding rapid prenatal counselling. [34] • Germline implications of cancer variants: Patients not referred for germline confirmation, due to lack of education/understanding of the genetic implications and lack of genomic expertise in the molecular tumour board [30] **Timeliness** was facilitated by the MDT approach, especially in the prenatal and acute paediatric scenarios: • Practical challenges and rapid turnarounds and genetic counselling ‘winging it’ due to urgent nature, lack of preparation, and time constraints often out of hours [31] • Time delays in patient referrals, testing, parental difficulty, poor communication, sample delays, plus impact on clinician time were noted as quite significant issues, and addressed by having more frequent MDTs and increased resourcing for lab processes [21] • The MDT helped facilitate rapid processes, collaboration and improved communications [32]	*‘MGTB interviewees indicated lack of education regarding medical genetics as a limitation of the MGTB, which is a recognized barrier to autonomous use of genetic testing by ordering physicians’* [30] *‘Genetics has traditionally been very nine to five, there’s no such thing as an emergency…’* [31]
	Clinician Behaviours within the MDT	**Acceptability:** Important role of MDT for bringing various medical disciplines together to solve complex cases [41]. A potential barrier if group dynamics became intimidating/power play of senior vs junior members [29] Also, if the MDT makeup does not include genetics/counselling expertise, then it is not an acceptable intervention due to potential risk to patients and inability to follow guidelines [30] **Adoption:** The MDT’s collaborative environment led to increased confidence in neurologists using genomics after MDT (66 to 94% change, p 0.004 [25] including helping to understand complexity, education on genomics and interpretation. Similarly in the acute setting, the MDT facilitated adoption of genomics [31]. A potential barrier of lack of genomic understanding amongst nephrologists including consent/counselling [27]	*‘High flexibility may be required by such a team, which needs to be aware of, and open to collaboration among different disciplines, different centres, different technologies, sometimes limited to remote-type of contacts and exchanges’* [22] *‘There’s a real, kind of, collaborative, multidisciplinary approach happening, and I think that’s really exciting.'* [31]
Outcomes	Health and Social Policy for MDTs	**Fidelity**: The MDT can become a ‘tick box’ exercise for enrolment due to increased demands for samples overriding clinical advice [29] **Sustainability:** The genomic MDT is resource intensive due to the need for careful discussion between disciplines, and this is not funded in normal clinical time [29]. In the acute setting, a rapid MDT team service needed standard operating procedures, lines of rapid communication and laboratory resourcing to be effective [21]. One study found a pattern of simpler cases being handled locally as their ability for genetic counselling improves, while the MDT service handles more complicated cases [35]	
	Economic utility of genomic MDT	**Cost**: The MDT was estimated to cost 399.61 pounds per case for discussion in meeting, 797 for sequencing, and 166.60 for analysis [41]. Other studies found an overall cost savings of a genomic MDT approach reaching diagnosis costing $2300 compared to an invasive renal biopsy costing $5300 [27]. Similarly, savings were introduced to the system by rapid genomic sequencing with efficiencies introduced by the MDT: Cost/diagnosis A$13388, additional savings 543 K in total cohort [21],however considerable demand on clinician time remained an issue.	

The healthcare contexts (Tables 1, 2) of the papers revealed a predominance of Western English speaking developed nations, including the United Kingdom (5 studies), North America (4 studies), Europe (3 studies) and Australia (5 studies). The clinical contexts were varied and included eight main areas: rare diseases (4 studies), prenatal genomics (4 studies), acute paediatric care (3 studies), renal (2 studies), and brain malformations, epilepsy, cardiac, cancer (1 study each). Most studies were conducted in Western specialised tertiary or national referral centres, with access to funded genomic testing and specialized expertise in subspecialty medicine and high degrees of genomic literacy. Only one study [21] utilized an implementation framework, and almost all (except for one qualitative study) exclusively focused on healthcare professionals as participants.

The quality scores for all studies were relatively low (average 0.5, range 0.2–0.7) due to a number of factors, especially in the lack of comparator, blinding, and relatively small sizes of the study cohorts. Some papers were more case reports [22] than research studies, and very few papers had a structured approach to the analytical methods, control for confounding, or assessment for bias.

### The MDTs role in facilitating genetic diagnosis including variants of uncertain significance

**Fig. 2:**
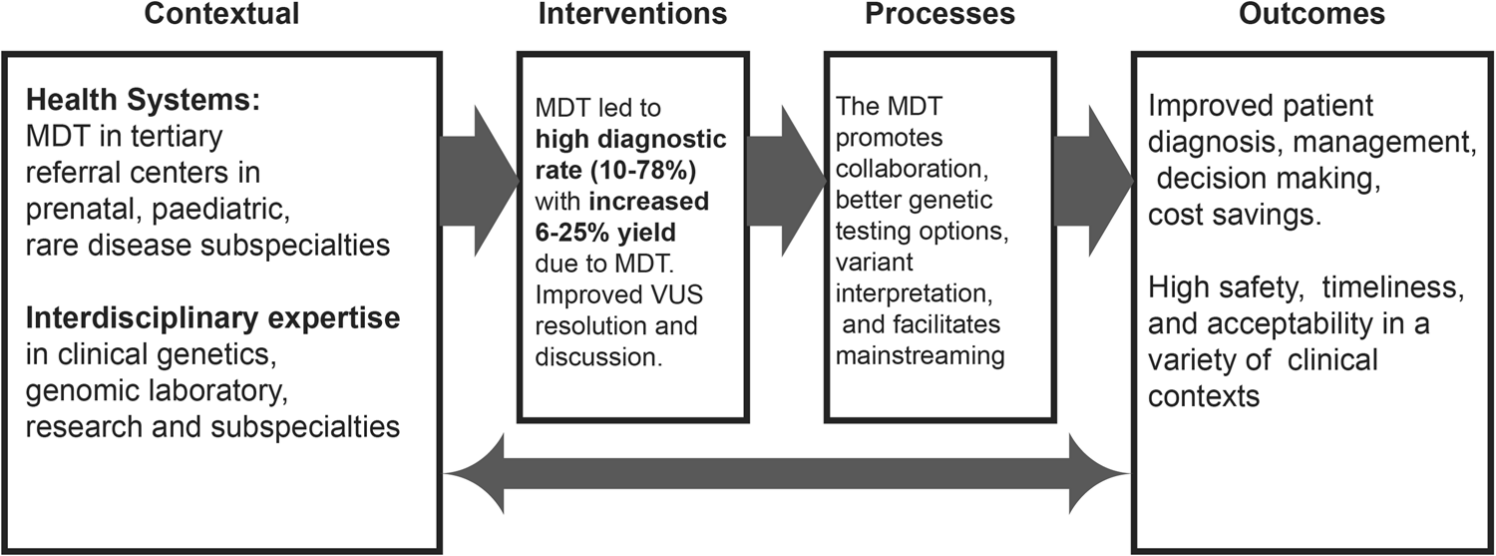
The genomic MDT in the GMIR framework. Summary of the main findings of the 17 studies, outlining the context, interventions, processes and outcomes of the genomic MDT.

The intervention of the genomic MDT was found to be effective and an efficient use of resources and expertise, requiring close collaboration and specialist clinical access to maximise diagnostic yield (Fig. 2, Table 2). All 17 studies pointed towards high effectiveness of the genomic MDT approach, utilizing interdisciplinary collaboration and expertise to achieve higher diagnostic yields in complex genomic cases (Fig. 2, Table 2). From the quantitative studies, genomic testing in the MDT context had a diagnostic yield of around 10-78% depending on the clinical contexts. Studies where the MDT’s role was examined specifically in this claimed an increased yield of around 6-25% additional to ‘standard’ testing (Table 2, Fig. 2).

The MDT’s role in efficient discussion and resolving of VUS was a significant theme, as these can be very complex and require detailed and time-consuming scientific and clinical correlation. The MDT approach led to VUS resolution by maximising the clinical-scientific interaction for better variant interpretation [23], preventing false positives [24], and reducing the overall number of variants requiring curation and time even up to 2/3 [25]. Others [26] highlighted the MDT’s role in providing more accurate genotype-phenotype correlation due to expert clinical inclusion; recommended re-examination of patients with possible syndromal features led to an ‘uplift’ in diagnosis in an additional 25% of patients in their small cohort of hypertrophic cardiomyopathy (HCM) being diagnosed [26]. The renal genomic MDT was essential for pre-test gene curation, leading to improved reports when the correct gene was reported and interpreted [27, 28].

While some studies examined the MDT’s role, many of these studies did not differentiate between having an MDT model in place or not, and therefore did not consider a ‘no comparator’ or control group in their design. This was mostly due to the MDT being integral to the actual study process, for example, including genomic sequencing and interpretation with an integrated MDT. These studies claimed a higher yield, using an MDT integrated approach due to the ability to maximise the collaboration between subspecialist expertise, laboratory and genetics in order to tackle the issues of triaging testing, careful selection of genes for analysis, timeliness, and clinical correlation of variants (Table 2).

### The MDT promotes collaboration, improved patient management, and genomic mainstreaming

Apart from the impact on diagnostic yield and efficient resolution of VUS, the MDT was also found to have additional benefits for the healthcare system and clinicians. In the qualitative studies with semi structured interviews in the setting of general genomics [29], cancer [30], and acute care genomics [31], interdisciplinary collaboration was described as a major contributor to the MDT’s high degree of acceptability and adoption across these different clinical contexts (Table 2). This was highlighted in the acute care setting [31, 32], as well as the brain malformations study [22]. Beyond the increased diagnostic yield components, the MDT process of a clinically-focussed discussion between subject matter experts (eg. clinical, radiology, research, genomic) aided novel gene discovery, diagnostic improvement, and functional genomic options for patients (Fig. 2, Table 2).

The genomic MDT intervention appeared to have an immediate impact on patient management, by being able to inform decision making in acute situations such as in 57% of acute rapid exome sequencing (rWES) cases [21], for sick neonates in intensive care. In epilepsy, 92% of diagnoses had an immediate impact on clinical management [25]. In the renal clinic, having a MDT confirmed clinical diagnosis (34%) or reclassification (39%) of diagnosis aided management in 59% of patients who avoided additional invasive testing such as biopsies, or had better surveillance and treatment options[27]. This was quantified by direct cost savings such as $AUD2300 as the cost of sequencing, leading to avoiding invasive renal biopsies of up to double the cost. In the acute cohort an estimated total $AUD534K savings were made, while the cost was $AUD13,388 per diagnosis [21].

Overall, the role of the MDT was to achieve better resolution of genetic variants in the correct clinical context, with the collaboration and expertise of clinical and laboratory expertise, research and clinical subspecialists. One study directly interviewed MDT members regarding the function of the MDT, asking members about decision making and the factors that affected their genomic practice [29]. They found that the MDT functioned as a triaging/selection process for genomic sequencing, and was beneficial in this process especially for shared decision making, genomic outreach to other subspecialties, and education in non-genetics professions. These are often cited as key factors for successful ‘mainstreaming’ of genomics [33].

### The genomic MDT processes are safe, timely, and acceptable in a variety of clinical contexts

A number of service level outcomes were highlighted including increased safety and timeliness of processes via the MDT (Table 2). There were several negative examples of how the genomic MDT impacted patient safety. A prenatal study found that using commercial laboratories without a genomic MDT approach had an almost unacceptably high number of VUS, which affected patient management in the time-pressured prenatal scenario, with 33% of tests reporting one or more VUS [34]. Most of these VUS were considered by MDT review to be unrelated to the actual diagnosis. In a molecular tumour board study (MTB) [30], patients were not recognised as potentially harbouring germline cancer predisposing variants due to the lack of genetic expertise and representation on the MTB. Without adequate genetic representation, there was significant confusion about interpretation of results, role delineation, and ultimately poor patient management. Therefore, it appears that is not sufficient just to have multiple members of an MDT, but there is a need to have the correct subspecialty/expertise for the cases being discussed, or risk an inadequate interpretation or implementation of outcomes, risking patient harm.

The genomic MDT approach also improved timeliness of results in time-pressured situations such as acute genomics and prenatal scenarios. In the prenatal situation, this made a significant difference due to the need for precise and rapid results to guide Foetal Medicine Unit specialists and patient counselling requiring a team approach for consensus and advice. Interestingly, over a 10 year period, a pattern emerged [35] of more complex cases and results required an MDT tertiary centre review, while more ‘simple’ cases were increasingly being able to be handled locally, possibly demonstrating changing roles of centres with and without MDT access.

In the acute care genomic scenario, the initial barrier of timeliness improved when MDTs were changed from every 16 days to weekly, in response to an implementation science based qualitative study, with additional workforce and laboratory resourcing provided to meet tight deadlines [21]. This did challenge existing models in genetics that were very much based on office hours and requiring time for detailed genetic counselling [31]. In addition, the MDT was found to be a facilitator of rapid processes due to the collaborative communication nature of the approach, and this became a key component of one acute care service [32].

Further to these service/patient level outcomes, the genomic MDT implementation outcomes included high levels of acceptability and adoption, although there were some potential tensions raised especially in the role of junior vs senior members of the MDT team, and the need for efficiency and high volume of cases sometimes overriding the quality review and clinical decision making process. In the rare disease diagnostics context, the genomic MDT was found to have clear benefits, for adoption, but there were potential issues of sustainability due to the time and resources required for consent and discussion, often outside of the allocated time for standard clinical consultations [29]. Similarly, the genomic MDT model was found to be appropriate in the renal context due to improving genomic education of nephrologists [28]. High adoption was reported in the prenatal context due to faster and more accurate diagnostic yield [36] impacting management.

One study quantified the level of acceptability of genomic adoption by non-genetics healthcare professionals and showed a shift from 66 to 95% confidence in genomic testing (*p* = 0.004) which is a statistically and clinically significant result [25]. Qualitatively the neurologists reported that the MDT approach with genetics/lab/research helped improve their education, understand the complexities of genomic testing and variant curation, and interpretation, and also facilitated being able to make comprehensive plans for return of results to families and management. This is significant as often the main barrier to mainstreaming of genomics reported by non-genetic clinicians includes education, understanding, and interpretation of genomic results [33].

## Discussion

The genomic MDT approach appears to be highly effective and efficient, facilitating higher diagnostic rates and improved patient management. There were additional flow on effects of improved acceptability of genomics, facilitating education and mainstreaming into the non-genetics workforce. However, there still remain significance evidence gaps in the actual costs, sustainability, and equity of access to the genomic MDT. Also, a systematic implementation science based approach to the genomic MDT is required to ensure its adoption into different health contexts, and inform health policy and practice.

### The genomic MDT is a model for interdisciplinary collaboration

The interdisciplinary genomic MDT provides a model for genomic medicine that incorporates interdisciplinary collaboration, with the most effective teams having the relevant subspecialist, clinical genetics, genetic counsellor, and research/functional genomics expertise to maximise diagnostic yield and minimize VUS. This is particularly important, with some studies reporting as many as 36% of patients receiving VUS from genomic testing [37]. This has been found to cause higher levels of anxiety and distress in some patients, and even uncertainty over management decisions [38]. In addition, VUS often end up being reclassified, many as benign, but this takes a considerable amount of time both in terms of laboratory technicians reviewing the evidence and data, and for research such as functional genomics and databases to help reclassify [39]. These efforts to help VUS interpretation include international massive population genomic databases, collaborative forums to refine variant curation and interpretation, and functional genomic research including machine learning and AI. In contrast, many of the studies included in this review reported the reduction in VUS, without the need for resource and time intensive international databases or functional genomics research, simply by incorporating the collaboration of laboratory scientific and clinical subspecialty expertise.

### Genomic MDT cost, sustainability, equity and scale up

Although most studies have favourably portrayed the impact of the genomic MDT, there are a number of evidence gaps including the sustainability, costs, equity of access, and scale up of genomic MDTs. This has important implications for precision medicine, as genomics and advanced therapies continue to ‘mainstream’ into standard healthcare [40].

In terms of costs, only three studies analysed the actual cost or cost effectiveness of the MDT approach [21, 27, 41]. These studies highlighted costs savings to health systems by reaching rapid diagnoses, and reduced ‘diagnostic odyssey’ including invasive testing, but these are indirect costs not directly due to the MDT approach. Also, some issues were raised such as whether insurance would cover the cost of testing, raising equity and access barriers. None of these studies gave an accurate quantification of the exact costs, resources and personnel required in the genomic MDT. This evidence would more accurately reveal the resources required for such an approach, realising that for many services the MDT is not within ‘normal business’ and therefore not independently funded, unlike genomic testing and clinical services. However, there could be a potential positive role for MDT in helping to educate non-genetics clinicians so that simpler cases could be handled locally without MDT input, and saving resources for more complex/timely cases. More research is required to fully elucidate the costs of an MDT approach, and any potential savings to the healthcare system from this model.

Another evidence gap is sustainability, as MDTs often occurred in a time constrained environment, such as acute paediatrics and prenatal genomics. In these scenarios, the need for a rapid answer sometimes led to tensions and difficulties in feasibility for standard practice, such as the time required to consent and discuss cases with patients and families. While one purpose of the MDT was to educate/feedback to clinicians regarding results, and discuss complex issues, these were often laid aside for the sake of efficiency [29, 41]. Also there was a recognised ‘blurring’ between research and clinical work and the additional workload of the MDT, often funded by research, was putting constraints on clinical services [29]. The ongoing sustainability of such an approach, combined with cost data, would inform health systems planning and policy around the MDT.

### Implementation factors to consider for genomic MDT sustainability

There are some additional considerations for the successful implementation of a genomic MDT. Firstly, these studies almost exclusively in highly specialized Western tertiary centres, requiring access to genomic testing funding, expertise including subspecialists and genomics laboratories, and sometimes even functional genomics research access (Table 2, Supp. Table S3). This raises the issue of equity, diversity, and adaptability to less complex, primary care, and non-tertiary settings. These MDTs were embedded within Western healthcare systems with clear networks of referrals, expertise, laboratory access, and mostly English speaking. Most importantly, access and payment for genomic testing was assumed, whereas this is not always available pending insurance coverage, local availability and funding mechanisms, especially in developing countries. This excludes a large proportion of worldwide healthcare systems and populations, and limits the generalizability of these studies to developing countries and non-english speaking contexts. It also risks perpetuating pre-existing inequalities of access to genomics and research away from under-served populations, where arguably there is much greater clinical need [42]. Also, the clinicians involved were highly qualified experts, with high genomic literacy and resourcing. Where such staff were not available there were potential issues of safety [30].

Secondly, the genomic MDT is particularly relevant for genomic ‘mainstreaming’ models being proposed worldwide, such as Genomics England [43] and in Australian Genomics [44]. The MDT, with its concentrated expertise and collaborative model, could be a vital piece in the efforts to facilitate non-genetics healthcare professionals undertaking genomics, by providing an intervention that is genomics informed, collaborative, and educational. This was found in one study where the adult epilepsy genomic MDT improved clinician confidence to undertake genomics themselves, by experiential learning [25]. This model has potential for facilitation of mainstreaming at scale, and address some of the workforce gaps in genetics worldwide. However, the equity, access, and diversity issues must be addressed, in order to have fair and equal access to the benefits of precision medicine for all.

Finally, we identified another gap in the lack of patient and family data on satisfaction with the MDT approach. No studies directly asked patients and families whether they wanted a MDT approach, with multiple people discussing their case and genetic information. This has also been identified previously as a gap in the cancer MDT literature [10]. Privacy concerns, litigation factors, and consumer involvement are potential areas for future study, as there is no consumer voice in any of these studies especially in optimal discussion and disclosure of results, and the direct impact of the MDT on patients themselves.

### More implementation science based research is required in genomics and precision medicine

A major weakness to these studies were that they were mostly descriptive cohorts, without use of a robust framework, model, or theory for implementation. This can be said of the genomic literature in general, where only a small fraction (1.75%) of papers are in the field of implementation, thus limiting their utility and ability to transform research into clinical practice [45]. While qualitative and descriptive studies do have a key role in research and have uncovered many important aspects of the MDT, most studies reported solely on the genomic diagnostic yield, but provide no evidence on implementing the genomic MDT into ‘best practice’ in our complex health systems. Also, the quality scores of these studies were very low, particularly a few which were mainly case vignettes. Only one implementation science-based study took a systems approach [21] to address barriers to the acute paediatric MDT. Questions that still need answering include what the optimal makeup of a genomic MDT for maximum impact, cost effectiveness, the MDT processes of discussion, documentation, and followup, and the patient/consumer perspective on these. In addition, the secondary outcomes of the MDT could include improved collaboration, research, education, and ‘mainstreaming’ of genomics – and these need to be studied both in terms of effectiveness and the scalability and adaptability of these for precision medicine.

## Conclusion

While there are major gaps in evidence, the studies reviewed all point towards the benefits of the genomic MDT and a need for such an approach for more effective and efficient patient diagnosis and management. The MDT harnesses improved collaboration and discussion of complex clinical scenarios and genomic results (especially for VUS) for improved diagnostic rate and patient care. More quality research drawing on implementation science methods are needed to evaluate the full potential of genomic MDTs. Such research could propose how best to adaptation interventions for local use, study scalability and sustainability, and study the health systems factors that can enable scale up whilst promoting access and equity.

### Supplementary information

PRISMA Checklist

Supplementary Tables
