# Supplementary material for: What is the power of a genomic multidisciplinary team approach? A systematic review of implementation and sustainability
Source: Eur J Hum Genet. 2024 Feb 20;32(4):381–91. doi: 10.1038/s41431-024-01555-5 (PMC10999446; doi:10.1038/s41431-024-01555-5)
Supplement: Supplementary file 2 — Supplementary Tables [file 41431_2024_1555_MOESM2_ESM.docx]

**Supplementary Tables**

**Table S1: Search terms in Medline, PsycInfo and EMBASE**

MEDLINE AND PSYCINFO:

| 1. | genomic*.mp. |
| --- | --- |
| 2. | Genetic Testing/ |
| 3. | Genetics, Medical/ |
| 4. | Genomics/ |
| 5. | Rare Diseases/ |
| 6. | Whole Exome Sequencing/ or High-Throughput Nucleotide Sequencing/ |
| 7. | Whole Genome Sequencing/ |
| 8. | Patient Care Team/ |
| 9. | Multidisc*.mp. |
| 10. | Multi-disc*.mp. |
| 11. | Interdisciplinary Research/ |
| 12. | Interdisciplinary Communication/ |
| 13. | Clinical Decision-Making/ |
| 14. | Genetics, Medical/ or Genetic Counseling/ or Genetic Services/ |
| 15. | Disease Management/ |
| 16. | Evidence-Based Medicine/ |
| 17. | Disease Management/ |
| 18. | "Delivery of Health Care"/ |
| 19. | Treatment Outcome/ |
| 20. | Patient Reported Outcome Measures/ |
| 21. | Diagnosis/ |
| 22. | 14 or 15 or 16 or 17 or 18 or 19 or 20 or 21 |
| 23. | 1 or 2 or 3 or 4 or 5 or 6 or 7 |
| 24. | 8 or 9 or 10 or 11 or 12 or 13 |
| 25. | 22 and 23 and 24 |
| 26. | limit 25 to (english language and humans) |

EMBASE terms:

| 1. | whole genome sequencing/ |
| --- | --- |
| 2. | genomic*.mp. |
| 3. | genetics/ |
| 4. | medical genetics/ |
| 5. | genomics/ |
| 6. | rare disease/ |
| 7. | Multidisc*.mp. |
| 8. | Multi-disc*.mp. |
| 9. | Interdisc*.mp. |
| 10. | multidisciplinary team/ |
| 11. | shared decision making/ |
| 12. | interdisciplinary communication/ |
| 13. | genetic counseling/ |
| 14. | diagnosis/ |
| 15. | patient care/ |
| 16. | disease management/ |
| 17. | clinical evaluation/ |
| 18. | clinical outcome/ |
| 19. | diagnostic value/ |
| 20. | health care delivery/ |
| 21. | patient-reported outcome/ |
| 22. | 13 or 14 or 15 or 16 or 17 or 18 or 19 or 20 or 21 |
| 23. | 7 or 8 or 9 or 10 or 11 or 12 |
| 24. | 1 or 2 or 3 or 4 or 5 or 6 |
| 25. | 22 and 23 and 24 |
| 26. | limit 25 to (human and english language and (article or "review")) |

**Table S2. Data extraction and definitions for genomic and MDT interventions -**

**mapped to TiDiER, Proctor outcomes and GMIR**

| **Column** | **Details** | **Options and definitions** |
| --- | --- | --- |
| Study | Authorship | First author and year published |
| Study | Country |  |
| Study | Study Design | Qualitative, Quantitative, Blinded, retrospective, prospective, cohort |
| Study | Clinical Context | Subspecialty of medicine, health system context |
| Study | Study Period | Time period |
| Study | Imp. Framework used | If included implementation science approach |
| Participants | HCP and patient participants | Members of MDT, including patients and clinicians interviewed in qualitative studies |
| Participants | Healthcare Setting | Hospital, community, clinic, referral centre |
| Participants | Patient Setting | Clinical details, including numbers of patients |
| Intervention | Genomic intervention | Type of sequencing and details  Eg exome or genome sequencing |
| Intervention | Genomic components | Number of patients sequenced,  Subtypes, if any |
| Intervention | MDT intervention | Broad description of MDT – setting, role, aim |
| Intervention | MDT Components | Frequency of MDT  Data Collected on MDT, eg. interviews, process data |
| Comparator | Alternative to MDT | Usually ‘standard’ care, may not be used |

| **Proctor Level** | **Proctor Outcomes** | **Application to genomic MDT** |
| --- | --- | --- |
| **Healthcare Service** | **Efficiency** | MDT as an efficient use of resources, time, money, services |
|  | **Safety** | MDT’s role in ensuring patient and service level safety |
|  | **Effectiveness** | Impact of the MDT in terms of quantitative (diagnostic yield, improved rate of diagnosis) and qualitative (impact on service delivery) |
|  | **Equity** | Equal access and distribution of genomic MDT across services |
|  | **Patient Centeredness** | Meeting the needs and aims of the patient in genomic diagnosis and management |
|  | **Timeliness** | Ensuring acceptable timeframe between genomic testing and results |
| **Implementation** | **Acceptability** | Satisfaction with various aspects of the MDT – at an individual clinician level |
|  | **Adoption** | Uptake, utilization, intention to implement/try the MDT approach at an individual practice level |
|  | **Appropriateness** | Perceived fit, relevance, compatability, suitability of the MDT process |
|  | **Cost** | Financial impact, Cost effectiveness, cost benefit, for provider or institution |
|  | **Feasibility** | Actual fit or utility, suitability for everyday use, practicability, at an organization level in the short term |
|  | **Fidelity** | Delivered as intended, adherence to protocol |
|  | **Penetration** | Level of integration, spread, service access, organization level |
|  | **Sustainability** | Maintenance over the long term, continuation, durability, incorporation, integration, sustained use, routinization in organization |

| **GMIR domain** | **GMIR Subdomain** | **Application to the genomic MDT** | **Mapping to Proctor’s Outcomes** |
| --- | --- | --- | --- |
| **GMIR - CONTEXTUAL FACTORS** | **Healthcare systems** | Access to MDT, resources and infrastructure, setting of healthcare system and social determinants of populations accessing MDT | **PROCTOR: Equity** |
|  | **Clinician Factors** | Demographics, psychosocial, stress, satisfaction, resources/time, expertise, attitudes, knowledge and understanding of genomics | **Proctor: Satisfaction** |
| **GMIR - INTERVENTION** | **Genomic Yield** | Effectiveness of MDT in achieving diagnoses | **PROCTOR: Effectiveness** |
|  | **Genomic Characteristics** | MDT characteristics which aid effectiveness, such as efficiency, adoption, collaboration | **PROCTOR: Efficiency, Appropriateness** |
| **GMIR - PROCESSES** | **Healthcare system processes** | Safety, Timeliness of the MDT | **PROCTOR: Safety, Timeliness** |
|  | **Clinician Behaviours** | Acceptability and adoption of MDT for clinicians | **PROCTOR: Acceptability, Adoption** |
| **GMIR - OUTCOMES** | **Health and Social Policy** | Fidelity, sustainability of the genomic MDT | **PROCTOR: Fidelity, Sustainability** |
|  | **Economic utility** | Cost/value, cost effectiveness | **PROCTOR: Cost** |

**Table S3. Summarising Proctor and GMIR Outcomes in all studies**

| GMIR – MDT Contextual factors | | GMIR – Intervention Domain  & Proctor Service outcomes | | GMIR – Processes Domain  & Proctor implementation outcomes | | GMIR – Outcomes Domain  & Proctor Outcomes | |
| --- | --- | --- | --- | --- | --- | --- | --- |
| Healthcare Systems | **Clinician factors** | **Genomic yield** | **Genomic characteristics** | **Healthcare system processes** | **Clinician behaviours** | **Health and social policy** | **Economic utility** |
| Ormonroyd 2017 (35)  UK qualitative GM MDT for ES NHS- UK Health System wide MDT | 19 clinicians interviewed about meetings, case inclusion, issues, function of MDT over 10 months of monthly meetings. | **Effectiveness** – MDT beneficial for case selection/ triaging and for shared decision making, increasing genomic outreach and education. | **Appropriateness**:  tension between **Efficiency** (need to get through many cases) and equity (volume high low quality) vs patient centeredness (clinical vs research) |  | **Acceptability** – beneficial to all members, BUT tension/barrier of senior vs junior power dynamic | **Fidelity**–tension between high volume/ research vs clinical careful decision making  **Sustainability:** time for consent and discussion not in standard clinical practice |  |
| Fishler 2019 (37)  MGTB cancer Qualitative  Single site USA | 12 members MGTB over 13 months interviewed and notes reviewed (note: only one geneticist) | **Effectiveness** – not fully realised due to lack of genetics involvement in cancer MDT, led to issues with lack of genetics understanding amongst non-genetics members (oncologists/pathologists) | **Efficiency:**  MDT guidelines not followed due to lack of role delineation, confusion, lack of genetic expertise | **Safety** – expose patients to potential danger due to lack of germline testing - additional cancers and affected family members not offered screening despite indications for testing from results discussed in MDT | **Acceptability**: MDT not an acceptable intervention due to lack of genetics expertise/ involvement and  poor knowledge/ education about genetics | **Fidelity**: can’t follow guidelines properly if no genetics expertise in cancer MDT |  |
| Mancini 2021 (24)  Brain malformations MDT QUALITATIVE  Netherlands referral centre | 3 Case vignettes demonstrate role of MDT in brain malformations | **Effectiveness** – assisted diagnosis **note some quantitative data of 3.7% increase yield due to MDT eval. | **Efficiency**– MDT interaction between research/clinical/ genetics/ subspecialist expertise vital  **Appropriateness:** blurring of lines research vs clinical |  | **Acceptability** – importance of MDT for VUS resolution, functional/ research for diagnosis |  |  |
| GMIR – MDT Contextual factors | | **GMIR – Intervention Domain**  **& Proctor Service outcomes** | | **GMIR – Processes Domain**  **& Proctor implementation outcomes** | | **GMIR – Outcomes Domain**  **& Proctor Outcomes** | |
| Healthcare Systems | **Clinician factors** | **Genomic yield** | **Genomic characteristics** | **Healthcare system processes** | **Clinician behaviours** | **Health and social policy** | **Economic utility** |
| Lynch 2020 (36)  Acute care for genetic counsellors QUALITATIVE  Over 12 tertiary hospital sites | 16 genetic counsellors interviewed in 12 hospitals over 2 years | **Effectiveness**: evolving role of genetic counsellor, and tension between collaborative MDT model or genetics led, need for better role delineation and education | **Efficiency:**  collaborative approach vital in acute setting, and MDT with GC role in education and upskilling others | **Timeliness** – challenging and potential barrier of acute counselling in time pressured and lack of preparation in this setting | **Adoption**– collaborative MDT vital for adoption of acute genomics, especially with key role of genetic counsellors in leadership, education and upskilling non-genetics staff | **Sustainability** – role of MDT for other areas and non geneticists upskilling |  |
| Hill 2020 (28)  Acute rapid genomics service QUALITATIVE  Single site GOSH (UK) | 19 clinicians and 11 parents interviewed in major centre for issues in process of rapid genomics. | **Effectiveness** – yield 42%, 30% benefit for management | **Efficiency** - collaborative relationships established for better diagnostic yield |  | **Acceptability**– rapidity required MDT process with communication |  |  |
| Vadlamudi 2021 (25)  Australian epilepsy cohort Quantitiative and qualitative | 104 patients referred over 12 months, 66 tested  MDT with neurologist, genetics, lab, research team | **Effectiveness** – 17% yield, 82% impact on management | **Efficiency**: 29 VUS in 19 patients discussed via MDT, and 5 deemed reportable/ actionable  And 2/3 reduction in number of variants requiring curation after MDT review |  | **Acceptability and Adoption**: increased confidence for neurologists in using genomics 66 to 94% (P=0.004) and improved understanding of the complexity of variants, education, and interpretation |  |  |
| Taylor 2019 (21)  Genomics MDT UK QUANTITATIVE National service | 132 patients underwent MDT discussion over 10 months, | **Effectiveness** – yield 31.6% including 6.6% alternative diagnosis, and guiding management | **Efficiency**: only 74.5% approved cases were sequenced,  MDT role in pre-test triaging and workup, and genomic outcomes. | **Timeliness** - need for increased frequency MDT to meet demand | **Acceptability:** importance of MDT interaction between clinician and scientists  **Adoption**: MDT model informed broader NHS 100K project |  | **Cost**: ~400/ meeting |
| GMIR – MDT Contextual factors | | **GMIR – Intervention Domain**  **& Proctor Service outcomes** | | **GMIR – Processes Domain**  **& Proctor implementation outcomes** | | **GMIR – Outcomes Domain**  **& Proctor Outcomes** | |
| Healthcare Systems | **Clinician factors** | **Genomic yield** | **Genomic characteristics** | **Healthcare system processes** | **Clinician behaviours** | **Health and social policy** | **Economic utility** |
| Lazaridis 2016 (22)  Mayo clinic WES QUANT  Single Site | 82 patients referred to Mayo over 18 months, 75 met criteria, 51 ended up tested | **Effectiveness** - yield 15/51 (29%), MDT helped reclassify 6 VUS to pathogenic | **Efficiency:** MDT determined efficient for WES and reclassifying 6 VUS to improve diagnostic rate |  | **Adoption**: MDT initially setup for pre-test screen, became post-result discussion board too |  | **Cost** - $8K per patient, and 11/71 patients denied due to insurance |
| Marinakis 2021 (23)  Greek exome study QUANT | 400 patients referred for genetics exome cohort | **Effectiveness** – 53% yield, 85 novel variants – yield highest especially in dermatology and syndromal patients, driven by phenotype and MDT filtering approach | **Efficiency:** MDT vital in phenotype driven strategy due to clinical collaboration, and variant analysis | **Safety** – having close clinical correlation reduced risk of false positives | **Acceptability**: close MDT collaboration between phenotype and genotype in team had best yield |  |  |
| Jayasinghe 2021 (26)  Renal ES study QUANTITATIVE  4 sites – tertiary | 225 patients referred to renal genetics in 4 MDCs | **Effectiveness** : 80/201 39% yield,  34% confirmed clinical diagnosis, 28% clarify, 39% reclassify – higher yield due to MDT triage and variant interpretation | **Efficiency**: MDT approach has a higher yield due to careful patient selection and variant analysis, with important management implications | **Safety**: 59% added management implications including no invasive renal biopsy, surveillance/ treatment implications | **Adoption**: barrier includes lack of education amongst non-genetics renal physicians | **Fidelity**: lack of genomic education in nephrologists limited use of MDT | **Cost**: ES $2300 vs biopsy $2600-$5300 led to savings and less invasive |
| Mallett 2017(27)  Renal panel cohort QUANTITATIVE  Single lab | 140 patients referred to expert renal genetics lab | **Effectiveness** – diagnosis 58/135 (43%) v high in alport 81% and tubular 17% rate of VUS | **Efficiency**– higher rate from MDT due to careful gene selection and curation, and variant interpretation by MDT |  | **Appropriateness**: MDT good model fit for clinical/gene interpretation |  |  |
| GMIR – MDT Contextual factors | | **GMIR – Intervention Domain**  **& Proctor Service outcomes** | | **GMIR – Processes Domain**  **& Proctor implementation outcomes** | | **GMIR – Outcomes Domain**  **& Proctor Outcomes** | |
| Healthcare Systems | **Clinician factors** | **Genomic yield** | **Genomic characteristics** | **Healthcare system processes** | **Clinician behaviours** | **Health and social policy** | **Economic utility** |
| Lunke 2018 (29)  Acute care rapid WES QUANTITATIVE  2 tertiary sites | 40 patients referred in 2 hospitals for acute rWES  Implementation Science based review with CFIR | **Effectiveness** : 21/40 diagnosed 52.5% with 23 conditions, 57% changed management, 23% died | **Efficiency:** MDT model heavily relied upon for rapid model, but potential barriers due to labour intensive and time pressures, impact on clinician time demands | **Timeliness**: average 16 days MDT regularity to weekly, to improve timeliness of reporting results back | **Adoption**: Standard Operating Procedures for MDT developed | **Sustainability**: workforce/lab barriers, bottlenecks, increased demand, resourcing challenges to meet timelines | **Cost**: savings from rWES and MDT: A$13388/ diagnosis, $534K savings over cohort |
| Mone 2019 (30)  UK Prenatal QUANTITATIVE  Single service - tertiary | Review 256 patients referred to fetal genetic MDT over 10 years | **Effectiveness** – diagnosis in 47.7%, genetic in 43.2% with testing | **Efficiency** – high yield but more complex diagnoses over time requiring MDT for close collaboration between FMU and local genetics services |  | **Acceptability:** combined fetal/genetic MDT better yield for diagnosis | **Fidelity**: changing with time, simpler cases sorted locally and complex diagnoses in MDT |  |
| Chandler 2018 (31)  UK rapid exome skeletal prenatal QUANTITATIVE  Single site | Prenatal skeletal dysplasias suspected in 19 cases, 16 tested with rapid trio WES | **Effectiveness** : high yield 13/16 81%  MDT helped case selection, data interpret, fast turnaround, aid counselling and management | **Efficiency**: assist diagnosis, counselling, turnaround time for faster decision making | **Timeliness/Safety**: MDT vital for case selection and data interpretation for rapid high yield diagnostic rate, improving counselling and pregnancy management | **Acceptability**: MDT helped achieve rapid diagnosis for teams involved | **Sustainability**: cost and resource intensive approach flagged |  |
| Petrovski 2019 (32)  Rapid fetal WES trios QUANTITATIVE  Single site | 517 fetal cases referred, 234 tested as trio WES with structural anomalies | **Effectiveness** : 24 (10%) diagnosed  20% had VUS  complex require collab of MDT genetic, lab, counsellor, fetal imaging | **Efficiency**: complexity of cases and results required MDT approach of lab and clinicians to achieve diagnosis |  | **Acceptability**– MDT approach undertaken due to high complexity of cases and results requiring discussions | **Sustainability:** question of scalability due to complexity and limited expertise for MDT in other settings |  |
| GMIR – MDT Contextual factors | | **GMIR – Intervention Domain**  **& Proctor Service outcomes** | | **GMIR – Processes Domain**  **& Proctor implementation outcomes** | | **GMIR – Outcomes Domain**  **& Proctor Outcomes** | |
| Healthcare Systems | **Clinician factors** | **Genomic yield** | **Genomic characteristics** | **Healthcare system processes** | **Clinician behaviours** | **Health and social policy** | **Economic utility** |
| Cornthwaite 2022 (33)  Fetal WES/ QUANTITATIVE study | 90 exomes, 31 panels, retrospective chart review with MDT | **Effectiveness**: 23% yield | **Efficiency**: with 5-6% increased yield of VUS with MDT expert review | **Safety**: Commercial labs had much too high VUS rate and compromised diagnostic rate. |  |  |  |
| Rupp 2019 (34)  HCM sequencing study QUANTITATIVE  Single site | 42 patients referred with HCM, 36 tested with 28 gene panel and MDT review | **Effectiveness**: 28/36 (78%) diagnosed and MDT helped aid additional diagnoses in 5 patients. Also additional 9 patients (25%) solved via MDT process | **Efficiency:**  MDT enhanced diagnostic yield significantly especially in clinical-genotype correlation, re-examination of patient with syndromal features, and genotype interpretation |  | **Acceptability** - combined MDT better due to complexity and broad differential in childhood HCM |  |  |
